# Global Postural Re-Education Versus Deep Neck Flexor Activation on Chronic Nonspecific Neck Pain with Forward Head Posture

**DOI:** 10.3390/jcm15124833

**Published:** 2026-06-22

**Authors:** Huda B. Abd Elhamed, Esraa Ahmed Mohamed Ahmed, Enas Fawzy Youssif, Amr M. Yehia, Mohamed A. Abdel Ghafar, Safaa M. Elkholi, Shahesta Ahmed Osama

**Affiliations:** 1 Department of Physical Therapy for Musculoskeletal Disorders, Faculty of Physical Therapy, Cairo University, Giza 12613, Egypt; dr_huda_bader@cu.edu.eg (H.B.A.E.); enas.fawzy@pt.cu.edu.eg (E.F.Y.); 2 Department of Orthopedic Physical Therapy, Faculty of Physical Therapy, October 6 University, Giza 12585, Egypt; esraa.ahmed.pt@o6u.edu.eg (E.A.M.A.); amr.moustafa.pt@o6u.edu.eg (A.M.Y.); shahesta.ahmed.pt@o6u.edu.eg (S.A.O.); 3 Physical Therapy Program, Batterjee Medical College, Jeddah 21442, Saudi Arabia; 4 Department of Rehabilitation Sciences, College of Health and Rehabilitation Sciences, Princess Nourah bint Abdulrahman University, P.O. Box 84428, Riyadh 11671, Saudi Arabia

**Keywords:** neck pain, global postural reeducation, deep neck flexor activation, disability evaluation, forward head posture

## Abstract

**Background and Objectives**: Chronic nonspecific neck pain (NSNP) is among the most common musculoskeletal disorders. Global postural re-education (GPR) might be effective in decreasing neck pain (NP) and dysfunction and improving forward head posture (FHP) by recovering muscle chains and reducing postural alteration. Deep neck flexor activation (DNF) might also decrease NP and improve FHP by improving DNF endurance. This study aimed to compare the effects of GPR versus DNF activation on pain, dysfunction, FHP, and DNF endurance. **Materials and Methods**: Forty-six physiotherapy students with chronic NSNP participated in this non-randomized comparative study and were allocated into two equal groups based on their availability and preference regarding session duration. Group A underwent GPR exercises combined with active neck exercises, whereas group B received DNF activation in addition to active neck exercises. All participants were assessed pre- and post-intervention for pain intensity using a visual analog scale (VAS), neck disability using the Arabic version of the neck disability index (NDI), FHP via a photometric method with Kinovea software, and DNF endurance using pressure biofeedback. **Results**: A significant effect of both treatments was reported on reducing pain intensity, improving the FHP and enhancing the neck functional status with no substantial differences between both groups. A significant improvement in DNF endurance was observed in both groups, with substantially higher values between groups in favor of the DNF group. **Conclusions**: Both GPR and DNF activation exercises were associated with reductions in pain and improvements in neck disability among physiotherapy students with chronic NSNP and FHP. Also, both CVA and DNF endurance improved, with more improvement observed in DNF endurance in the DNF group compared with the GPR group.

## 1. Introduction

Chronic nonspecific neck pain (NSNP) has been identified in the literature as the fourth most common cause of disability worldwide, with annual prevalence rates of more than 30% [1,2]. Neck pain (NP) can be specific or nonspecific. Specific NP is caused by trauma or degenerative diseases, and NSNP is characterized by NP with an unclear cause that occurs when the same posture is repeatedly adopted or maintained for prolonged periods [3]. NSNP is defined as chronic if it persists for more than 3 months [4].

Physiotherapy students experience a high prevalence of NSNP, with episodes ranging from 34.6% to 54.8% [5]. These episodes may be attributed to factors such as manual handling of cases during clinical training, extended working hours, prolonged use of smartphones and laptops, backpack carrying, and poor posture, all of which promote increased forward head posture (FHP), which is characterized by an increased craniocervical angle beyond 50 degrees, and inhibition of the deep neck flexors (DNF) [6,7]. NP has been significantly correlated with FHP. Evidence from prior studies reveals that about 60% of individuals with NP display FHP [8].

The DNF muscles play an essential role in maintaining cervical lordosis [9]. NP, which results in muscular insufficiency, has been linked to reduced activation of DNF muscles in previous studies involving individuals with cervical impairment [10]. This reduction in activation may contribute to altered head and neck posture. FHP has also been associated with neck discomfort [8]. However, the role of DNF training in individuals with NP and FHP remains controversial.

Therapeutic strategies such as low-load exercises combined with biofeedback training are commonly used to activate DNF muscles, particularly longus capitis and longus colli [11]. Previous studies have shown that activating these muscles can reduce neck pain and improve functional disability [12,13].

Biofeedback training combined with DNF exercises enhances motor behavior by encouraging patients to engage in goal-oriented behavior [14]. Furthermore, it can strengthen the sensory feedback mechanism by providing visual cues that modulate motor unit discharge frequency and recruitment, ultimately resulting in improved DNF training outcomes [15,16].

Global postural re-education (GPR) is a physiotherapy approach based on the concept that muscles work as interconnected chains that may become shortened or imbalanced due to poor posture, repetitive activities, musculoskeletal disorders, or psychological stress [17]. GPR aims to restore muscle balance and improve postural alignment through global stretching, breathing control, and postural correction exercises. To accomplish this, it utilizes prolonged global stretching postures targeting muscle chains, performed with active patient involvement. The therapeutic sessions include sequential postures and gentle active movements that target muscle lengthening, joint decompression, and realignment, along with breathing control, antagonist muscle contractions, and sensory integration exercises, to reinforce proprioceptive input and improve postural control [18].

To the best of the authors’ knowledge, only a few studies have compared GPR with DNF-specific neck exercises for pain and disability in patients with neck pain [4,19]. In addition, no study to date has explored their effects on FHP and DNF endurance [17]. This gap in the literature raises an important clinical question: Does GPR produce greater improvements in pain, disability, FHP and DNF endurance compared to DNF activation in patients with chronic NSNP exhibiting FHP?

## 2. Materials and Methods

### 2.1. Study Design

This study was designed as single-blinded non-randomized comparative intervention study. It was conducted at the outpatient physical therapy clinic of October 6 University between December 2024 and April 2025. Participants were allocated to treatment groups based on their time availability. Pre- and post-intervention assessments were carried out by an independent blinded assessor.

The study was prospectively registered at ClinicalTrials.gov (NCT06711497) on 29 November 2024. Ethical approval was obtained from the Review Board of the Faculty of Physical Therapy, Cairo University (P.T.REC/012/005365) on 19 September 2024, and written informed consent was obtained from all participants, including consent for the use of anonymized photographs for publication.

**Figure 1:**
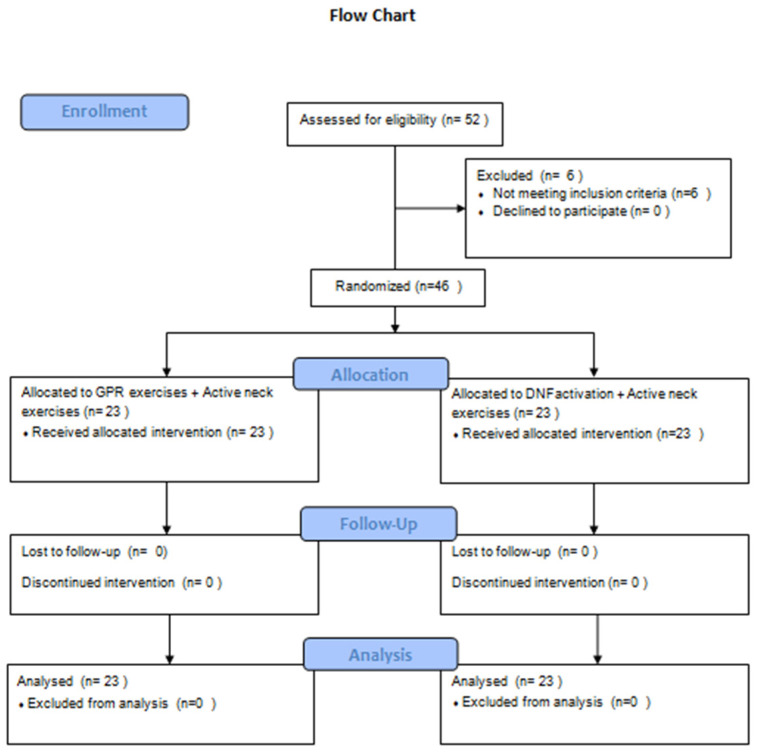
The flow diagram of the participants through the stages of the study.

This study followed transparent reporting principles for non-randomized intervention studies in accordance with the TREND statement [20]. The completed TREND checklist is provided in the Supplementary Materials (Supplementary Table S1). Participant recruitment and allocation are illustrated in Figure 1.

### 2.2. Sample Size

The sample size was estimated using G*Power software (version 3.1.9.2) based on an F-test for a repeated-measures MANOVA with a between-groups factor. The calculation was performed using the neck disability index (NDI) as the primary outcome measure. Assuming an effect size of 0.33, a significance level (α) of 0.05, and a statistical power of 80%, the analysis indicated that a minimum total sample of 44 participants was required to detect statistically significant differences between groups.

### 2.3. Participants

A total of forty-six physiotherapy students of both sexes diagnosed with chronic NSNP participated in this study and were assigned to two equal groups. Participants in Group A performed GPR exercises in addition to active neck exercises, while those in Group B received DNF activation combined with the same active neck exercise program.

Participants were eligible if they were physiotherapy students aged 18–24 years and had a history of chronic NSNP persisting for at least three months. Additional criteria included a minimum score of 3/10 on the visual analog scale (VAS) [21], a neck disability index (NDI) score exceeding 20% [22], the presence of FHP defined by a craniovertebral angle of less than 50° [23], and a body mass index (BMI) ranging from 18.5 to 24.5 [24].

Participants were excluded if neck pain was attributed to specific pathological conditions such as rheumatic or systemic neuromuscular disorders. Other exclusion criteria comprised cognitive impairment, cervical radiculopathy, cervical spondylolisthesis, or a history of head or neck surgery. Individuals presenting with additional musculoskeletal complaints, including lumbar or knee pain, were also excluded. Furthermore, participants were required to have not received any concurrent physical therapy or medical interventions, including electrotherapy, therapeutic exercises, analgesics, or anti-inflammatory medications, prior to enrollment [25].

Participants were allocated to the intervention groups based on their availability and preference for session duration. Specifically, individuals who were able to attend longer sessions were assigned to Group A (GPR exercises), which required approximately one hour per session, whereas those with limited time availability were assigned to Group B (DNF activation), with sessions lasting approximately 30 min. In addition to the primary intervention, both groups received a standardized program of active neck exercises as part of the rehabilitation protocol.

### 2.4. Procedures

#### 2.4.1. Assessment Procedures

After collecting demographic data, participants underwent a comprehensive baseline assessment of pain intensity, neck disability, and craniovertebral angle. Pain intensity was evaluated using a VAS, neck disability was assessed using the Arabic version of the NDI, and craniovertebral angle was measured using Kinovea software (version 2023.1.2; Kinovea Open Source Project; www.kinovea.org). All outcome measures were evaluated at baseline (pre-intervention) and after 4 weeks of intervention (post-intervention). To minimize assessment bias, the evaluators were blinded to the participants’ group assignments, ensuring that the measurements remained objective and unbiased throughout the study.

##### Pain as Measured by VAS

Pain intensity was assessed using a VAS, a widely accepted instrument with established reliability (0.60–0.77) and validity (0.76–0.84) [26]. The VAS consisted of a 100 mm horizontal line anchored by “no pain” at one end and “worst imaginable pain” at the other. Participants were instructed to indicate their current pain level by marking a point along the line [27].

##### Neck Disability as Measured by NDI

Neck disability was assessed using the Arabic version of the NDI (NDI-Ar). The NDI is a well-established instrument with documented moderate validity (0.50–0.70) and acceptable test–retest reliability [28], while the Arabic adaptation has shown excellent psychometric properties, including high reliability (ICC = 0.96), strong concurrent validity (0.92), and a two-factor construct explaining 67.58% of the variance [29]. The NDI-Ar comprises 10 self-reported items covering pain intensity and daily functional activities. Each item is scored on a scale from 0 to 5, yielding a total score out of 50, which is commonly converted into a percentage to facilitate interpretation [30].

##### FHP (CVA) Using Kinovea Software

FHP was quantified using the craniovertebral angle (CVA) through a photometric method. This approach provides a simple and objective assessment of postural alignment and has demonstrated high reliability, with inter-rater ICC values exceeding 0.972 and intra-rater values above 0.774. CVA measurement in the sagittal plane has also shown excellent reliability (ICC = 0.98) [31]. Digital images were captured and analyzed using Kinovea software, which has been validated for postural assessment with inter-rater reliability ranging from 0.95 to 0.98 and intra-rater reliability from 0.98 to 0.99 [32].

For image acquisition, a 64-megapixel mobile camera was mounted on a tripod at a distance of 150 cm and adjusted to shoulder height. Anatomical landmarks, including the tragus of the ear and the spinous process of the seventh cervical vertebra (C7), were identified using adhesive markers. Participants stood in a relaxed lateral position with weight evenly distributed and gaze directed forward. They were instructed to perform three cycles of cervical flexion and extension before assuming a natural head posture. A photograph was then captured, and the CVA was calculated as the angle formed between a line connecting the tragus to C7 and a horizontal line passing through C7 [33,34,35].

##### Deep Neck Flexors Endurance as Measured by Pressure Biofeedback

**Figure 2:**
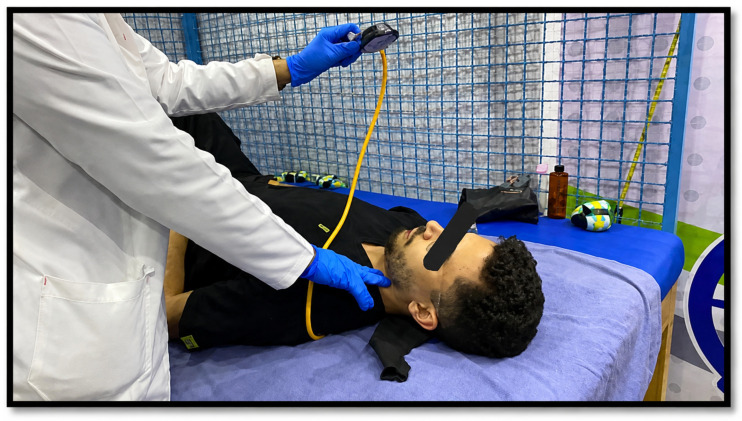
Evaluation of deep cervical flexor endurance using a pressure biofeedback device.

Deep neck flexor endurance was assessed using a pressure biofeedback unit. The device consists of an inflatable pressure sensor connected to a manometer, allowing quantification of muscle activation. The unit was positioned beneath the occiput between the cervical spine and the supporting surface and inflated to a baseline pressure of 20 mmHg. Participants performed a gentle cranio-cervical flexion movement (“nodding” action), progressively increasing pressure in increments of 2 mmHg up to 30 mmHg (22, 24, 26, 28, and 30 mmHg). Each level was maintained for 10 s. The test was terminated when the participant failed to sustain the target pressure for the required duration or successfully completed the highest level [36]; see Figure 2.

#### 2.4.2. Treatment Procedures

Both groups performed a standardized program of active neck and shoulder exercises. Cervical range of motion (ROM) exercises were carried out in all directions, including flexion, extension, bilateral rotation, and lateral flexion. In addition, active shoulder movements included flexion and abduction up to 90°, as well as internal and external rotation performed with the elbows flexed at 90° and the arms maintained close to the body.

All exercises were conducted in a seated position, and participants were instructed to perform movements within a comfortable range without holding the end position. A standing shoulder shrug exercise was also incorporated. Furthermore, participants performed a closed-chain upper limb activity by sitting on a chair, placing their hands behind their thighs, and lifting their body using upper limb support.

Each exercise and movement direction was executed five repetitions in the initial session, with progression to 20 repetitions achieved over four-week program course. Participants were specifically instructed not to sustain the end position of any active ROM exercise [37].

Group (A)

Participants in this group were trained on GPR exercises in addition to active neck exercises. Each session included two different lying postures to stretch the anterior and posterior muscle chains and one standing posture to integrate postural correction into activities of daily living (ADL). Participants initially adopted a supine position to minimize gravitational load, maintaining a posture directed toward elongation of the anterior muscle chain for approximately 15 min. This was followed by a second supine posture emphasizing the posterior muscle chain for an additional 15 min. The session concluded with a 5 min standing posture to promote postural integration under gravity load. Short rest breaks were provided between postures. The intervention consisted of two sessions per week over a four-week duration [38].

A—Stretching of the anterior muscle chain

In the initial phase, participants assumed a supine position with the upper limbs abducted to approximately 45°. The hips were positioned in flexion, abduction, and external rotation, with the plantar surfaces of the feet maintained in contact. This posture aimed to lengthen the anterior muscular chain, including the diaphragm, pectoralis minor, scalene muscles, sternocleidomastoid, intercostals, iliopsoas, and upper limb flexor groups.

The exercise began with regulation of breathing, focusing on coordinated activation of the diaphragm and accessory respiratory muscles. Proper alignment was established through gradual elongation of the lower limb musculature, followed by segmental adjustment using low-intensity isometric contractions applied in progressively lengthened positions. This approach facilitated post-isometric relaxation and improved postural awareness. Alignment was maintained through continuous verbal instructions and manual guidance provided by the therapist. During this phase, the lower limbs were progressively extended while preserving the corrected alignment [39].

**Figure 3:**
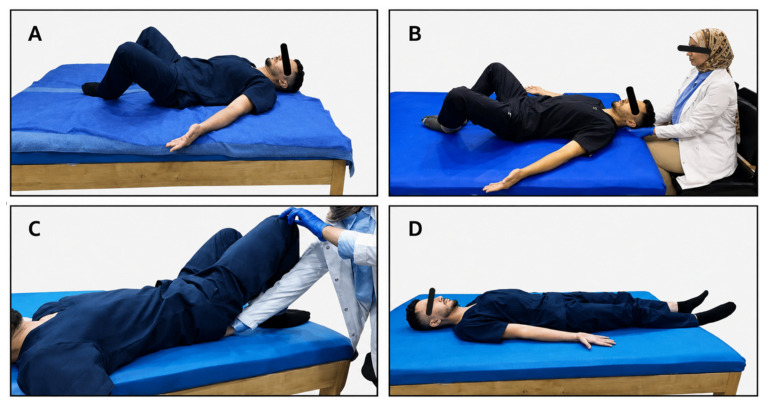
Sequential progression of anterior muscular chain stretching and postural alignment correction. (**A**) Initial posture: Supine position with upper limbs at 45° abduction, hips flexed/abducted, and plantar surfaces in contact to target the anterior chain muscles. (**B**) Manual traction: Therapist applying gentle manual traction at the occipital region to assist spinal alignment alongside rhythmic expiratory breathing. (**C**) Segmental adjustment: Therapist providing manual guidance and low-intensity isometric contractions to progressively extend and align the lower limbs. (**D**) Final progression: Achievement of full lower limb extension with neutral rotation, and upper limbs returned toward a neutral position to complete the postural alignment.

Progression of this posture involved the application of gentle manual traction at the occipital and sacral regions to assist spinal alignment. As control improved, hip positioning was gradually modified by increasing abduction and external rotation, followed by progression toward extension, adduction, and neutral rotation. Simultaneously, the upper limbs were guided from 45° abduction toward a neutral position to increase the stretch on the superior shoulder girdle musculature. Throughout the posture, subjects performed deep, rhythmic expiratory breathing; see Figure 3.

B—Stretching the posterior muscle chain

For the posterior chain, participants remained in a supine position with appropriate stabilization of the head, lumbar region, and sacrum. The hips were flexed to 90°, and gradual knee extension was performed to lengthen the posterior musculature, including the upper trapezius, suboccipital muscles, levator scapulae, erector spinae, hamstrings, triceps surae, gluteus maximus, and intrinsic foot muscles. Alignment was achieved using a similar sequence of progressive muscle elongation and low-intensity isometric contractions, aiming to promote neuromuscular control and postural correction. Manual facilitation and verbal feedback were used to support accurate positioning and ensure active engagement. A combination of sustained stretching, reflex facilitation, and gentle manual distraction at the occiput and sacrum was applied to optimize muscle length and alignment. The lower limbs were gradually extended as tolerated while maintaining postural corrections.

**Figure 4:**
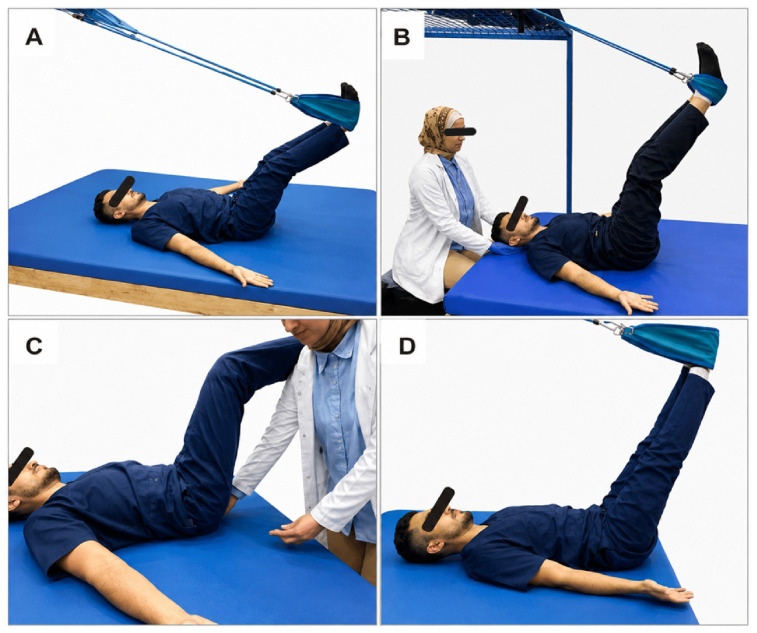
Sequential progression of posterior muscular chain stretching and postural alignment correction. (**A**) Initial posture: Participant in a supine position with the hips flexed toward 90° and feet supported in suspension slings to initiate gradual knee extension and target the posterior musculature. (**B**) Manual traction: Therapist applying gentle manual distraction at the occiput to assist spinal alignment alongside deep, rhythmic expiratory breathing. (**C**) Segmental adjustment: Therapist providing manual facilitation, sacral guidance, and low-intensity isometric contractions to ensure proper pelvic and lower limb alignment. (**D**) Final progression: Advanced positioning with progressive knee extension, ankle dorsiflexion, and incremental adjustments toward neutral hip rotation to maximize the posterior chain stretch.

Progression included the application of traction at the occiput and sacrum, followed by incremental adjustments in hip position toward adduction, flexion, and neutral rotation. Knee extension and ankle dorsiflexion were also advanced, while the upper limbs were guided into increasing degrees of adduction. Throughout each position, participants were instructed to maintain deep, rhythmic expiratory breathing [38]; see Figure 4.

C—Global postural re-education from standing position

In the final phase of treatment, participants assumed a standing posture to facilitate incorporation of postural adjustments into routine functional activities. During global stretching, postural compensations related to excessive tension in specific muscle groups were carefully avoided, and participants were encouraged to maintain smooth, unheld breathing. The session concluded with participants maintaining an upright stance for 5 min.

Progression in this position included gradual adjustment of lower limbs into adduction, extension, and neutral rotation, accompanied by gentle manual traction applied at the occiput to support axial elongation. The feet and toes were positioned in normal alignment with the floor. The shoulder joints were similarly guided into progressive adduction while being maintained in neutral rotation. Deep rhythmic expiratory breathing was required throughout.

Group (B)

Participants in this group performed DNF activation using pressure biofeedback in addition to active neck exercises. The pressure sensor was positioned beneath the neck, and participants performed a gentle nodding action, as if saying “yes,” to increase pressure by 2 mmHg above the 20 mmHg baseline, then by 4, 6, 8, and 10 mmHg sequentially without rest. By the end of movement sequence, the sensor was expected to register 30 mmHg [32].

Each pressure increment was maintained for approximately for 2 s, 10 s total after all 5 increments. The highest pressure level achieved with correct form was repeated for 10 repetitions with 10 s holds, performed in three sets with 30 s rest intervals between sets. The intervention was administered twice weekly over a period of four weeks [40].

### 2.5. Statistical Analysis

Statistical analyses were conducted using SPSS for Windows, version 26 (SPSS, Inc., Chicago, IL, USA). Before performing the primary analyses, data were assessed for normality, homogeneity of variance, and extreme values. Normality was assessed using the Shapiro–Wilk test, while homogeneity of variance was examined using Levene’s test. The significance level was set at *p* < 0.05.

Descriptive statistics were presented as mean ± standard deviation for continuous variables and frequencies and percentages for categorical variables. Independent sample *t*-tests were used to compare demographic characteristics between groups, while Chi-square tests were applied for sex distribution.

A repeated-measures MANOVA was conducted to examine the effects of time (pre- and post-intervention) and group × time interaction on pain intensity (VAS), functional disability (NDI), deep neck flexor endurance (DNF), and craniovertebral angle (CVA). Effect sizes were reported using partial eta squared (η^2^p), to indicate the practical relevance of the findings.

## 3. Results

Forty-six subjects with chronic NSNP were included in the current study. Participants were subdivided into two groups; Group A (*n* = 23) received GPR, and Group B (*n* = 23) received DNF muscle activation. The dependent outcomes were measured pre-intervention (pre) and at the end of the treatment intervention after 4 weeks post-rehabilitation (post).

The distribution of females and males in the GPR group was 60.8% (*n* = 14) and 39.2% (*n* = 9) respectively, while in DNF group, it was 65.2% (*n* = 15) and 34.8% (*n* = 8) respectively. Comparison of sex distribution for all participants in both groups using the Chi-square test revealed no statistically significant difference in sex distribution between groups (χ^2^ = 0.09, *p* = 0.76).

An independent sample *t*-test was conducted to examine differences in demographic characteristics between groups. The analysis showed no significant differences in height (*p* = 0.113), weight (*p* = 0.280), and BMI (*p* = 0.881). However, a statistically significant difference in age was observed between the two groups (*p* = 0.024). This difference may indicate a baseline imbalance between groups. This baseline age difference has been considered during interpretation of the findings. Detailed descriptive statistics and between-group comparisons are presented in Table 1.

### Repeated Measures MANOVA

Repeated measures MANOVA were conducted to investigate the effects of time and group × time interaction on pain intensity (VAS), functional disability (NDI), DNF endurance, and CVA.

A statistically significant group × time interaction effect was observed for DNF (F = 11.071, *p* = 0.002), indicating differential changes between groups over time. No significant interaction effects were found for VAS (*p* = 0.240), NDI (*p* = 0.576), or CVA (*p* = 0.614).

A significant main effect of time was identified for all dependent variables (*p* < 0.001), indicating improvement across both groups regardless of the intervention type. Large effect sizes were observed for the main effect of time across all dependent variables, indicating substantial improvements following the interventions. These findings are presented in Table 2.

A significant reduction in pain intensity and improvement in functional ability were observed in both groups following rehabilitation compared with pre-intervention measures. Furthermore, significant improvements were observed in DNF endurance and CVA. However, between-group comparisons showed no significant differences in most outcomes, except for post-rehabilitation DNF function, which demonstrated a significantly greater improvement in one group; see Table 3.

## 4. Discussion

This study examined the comparative effects of GPR and DNF activation on pain, disability, FHP, and DNF endurance in individuals with chronic NSNP. The findings demonstrated that both interventions were associated with significant improvements in all measured outcomes. However, the DNF activation group exhibited greater improvement in DNF endurance, while no significant differences were observed between groups in pain, disability, or craniovertebral angle (CVA). Given the non-randomized design and unequal intervention duration, these between-group findings should be interpreted cautiously.

### 4.1. Pain

Both GPR and DNF activation resulted in significant reductions in pain, with no significant difference between the two interventions. These findings indicate that both interventions were associated with clinically meaningful improvements in individuals with chronic NSNP.

The pain alleviation after GPR may be attributed to its effect on muscle chain rebalancing, where shortened superficial muscles are elongated and deep stabilizing muscles are activated. This reduces abnormal mechanical loading on cervical structures as well as nociceptive input. Additionally, the use of diaphragmatic breathing exercises could improve relaxation and autonomic regulation which helps reduce pain [2,16].

Previous studies have reported favorable effects of GPR in reducing pain and disability improvement compared to manual therapy [39,41,42]. Also, another meta-analysis reported moderate improvement in pain and dysfunction among individuals with spinal disorders, which support our results that GPR is an effective intervention for managing chronic NSNP [43]. In contrast, some studies suggest that its benefits may not be higher than other interventions. For instance, certain studies reported that both GPR and neck-specific exercises produced similar improvements in pain, disability, and neck mobility in women with chronic NSNP, with no notable variation between the groups [3,44].

Similarly, several recent studies have demonstrated that DNF activation exercises lead to significant reductions in pain for individuals with NSNP. The mechanisms behind this improvement may involve enhanced cervical segmental stability and neuromuscular control, decreasing compensatory overactivity of superficial muscles and mechanical stress in the neck [10,45]. These results are consistent with increasing evidence that DNF activation is an effective intervention for NP management. These findings support the clinical relevance of incorporating DNF activation into rehabilitation programs aimed at reducing pain in individuals with NSNP [46,47,48].

While DNF activation significantly reduced pain in most trials, some studies claimed that DNF training only addresses one aspect of NSNP, while others concentrated on ROM and muscle strength assessment with pain reduction as a secondary observation. Others still focused more on functional and postural benefits. It is necessary to consider other factors, including psychological, occupational, and general musculoskeletal concerns. Therefore, multimodal therapies may be required to achieve optimal outcomes [49,50].

### 4.2. DNF Endurance

This investigation found that both GPR and DNF activation exercises were associated with significant improvements in DNF endurance, with greater improvement observed in the DNF group. However, this between-group difference should be interpreted cautiously given the non-randomized allocation procedure and unequal intervention duration.

The observed improvements may be attributed to sustained postural holds, proprioceptive training, and neuromuscular re-education, which may facilitates tonic activation of DNF muscles. This aligns with reports indicating that DNF-specific training more effectively improves neuromuscular coordination and endurance than general or global programs [45,51].

Previous studies showed that DNF activation exercises resulted in greater improvement in DNF endurance and conflict on improving CVA [10]. A systematic review indicated that DNF-specific exercises produce greater improvements in endurance compared to general or global exercise approaches [52].

In disagreement with our findings, some studies suggest that GPR alone may not fully optimize DNF performance unless combined with additional feedback or training tools [53]. One study compared GPR with neck-specific exercises and it showed that both GPR and neck-specific exercises had similar results in improving DNF endurance [3].

### 4.3. Forward Head Posture (CVA)

Several methods have been used to assess postural alignment and forward head posture, including photogrammetry, digital image analysis, three-dimensional motion analysis systems, wearable sensors, and inclinometer-based measurements. Among these approaches, photogrammetric assessment of the craniovertebral angle is widely used because it is non-invasive, cost-effective, and demonstrates acceptable reliability for evaluating forward head posture [54]. The present study employed digital image analysis using Kinovea software to quantify changes in craniovertebral angle.

This study found that both GPR and DNF were associated with improvements in CVA in people with chronic NSNP and FHP without any significant difference between the two interventions in terms of CVA. Given the absence of a significant between-group difference and the methodological limitations of the present study, these findings should not be interpreted as evidence that the two interventions are equivalent for improving postural alignment.

The improvement following GPR can be attributed to global postural realignment, which restores balance between anterior and posterior muscle chains [42,53,55]. On the other hand, DNF activation contributes to improved CVA through enhanced deep cervical stabilization, reducing forward translation of the head [46]. These findings are in agreement with previous studies reporting improvements in CVA following both GPR and DNF activation exercises [17,52]. However, evidence remains inconclusive, as some studies report improvements in endurance without significant postural correction, highlighting variability in outcomes depending on intervention design [56].

### 4.4. Disability

In this study, both GPR and DNF activation exercises were associated with significant improvements in disability among individuals with chronic NSNP and FHP, with no significant difference observed between the two interventions, consistent with trials and systematic reviews reporting that both GPR and DNF-oriented or neck-specific exercise programs reduce disability and improve function in chronic NSNP [2,45,51].

The positive effect of DNF activation exercises on disability may be attributed to increased cervical stability and neuromuscular control, which decreases mechanical stress and optimizes functional performance. Previous systematic reviews and meta-analyses have reported that exercises targeting the deep cervical flexors are effective in reducing disability and improving function in individuals with chronic NSNP [52,57,58].

However, the degree of improvement in disability and function varied across studies, and the overall quality of evidence was classified as moderate to low, indicating the need for more high-quality, standardized research [53].

Although both interventions were associated with favorable outcomes, the present findings should not be interpreted as evidence of equivalence or superiority because the study was non-randomized and intervention dosage differed between groups.

The findings of the present study suggest that both GPR and DNF activation exercises were associated with improvements in pain intensity, functional disability, craniovertebral angle, and DNF endurance in individuals with chronic nonspecific neck pain and forward head posture. These findings may assist clinicians when selecting exercise interventions according to patient needs, treatment objectives, and available clinical resources.

This study has some limitations. First, the intervention dosage was unequal between groups, as GPR sessions lasted approximately twice as long as DNF sessions. This difference may have independently influenced treatment response through increased therapist interaction, exercise exposure, or patient engagement. Therefore, direct comparisons regarding intervention superiority should be interpreted cautiously. Second, group allocation was not randomized but was based on participants’ availability and preference regarding session duration. Consequently, baseline equivalence between groups may not have been fully achieved, which could limit the internal validity of the study. Additionally, the significant baseline age difference between groups should be considered when interpreting the findings. In addition, unmeasured factors such as motivation, schedule flexibility, treatment adherence, and lifestyle characteristics may have influenced the outcomes. Therefore, the findings should be interpreted cautiously, particularly regarding between-group comparisons.

Furthermore, the absence of a structured home exercise program may limit the generalizability of the findings to routine clinical practice, where home exercises are commonly prescribed. Future studies should examine whether incorporating structured home exercise programs alongside clinic-based sessions can further improve pain, disability, forward head posture, and deep neck flexor endurance outcomes. Moreover, the absence of a control group makes it difficult to definitively isolate the effects of the interventions from natural recovery, placebo effects, or other nonspecific factors. Consequently, future randomized controlled trials including a control group are recommended. Future studies should also adopt randomized designs with standardized intervention durations while following CONSORT or TREND reporting recommendations where appropriate.

## 5. Conclusions

GPR and DNF activation were both associated with reductions in pain intensity, improvements in functional disability, and enhancements in CVA and DNF endurance in physiotherapy students with chronic NSNP and FHP. The DNF activation group demonstrated greater improvement in DNF endurance compared with the GPR group. However, because of the non-randomized design and unequal intervention durations, causal inferences and superiority claims should be interpreted cautiously.

## Figures and Tables

**Table 1:** Descriptive statistics and independent sample *t*-test for demographic characteristics of participants.

Variable	Group A (GPR) N = 23	Group B (DNF) N = 23	t-Value	*p*-Value	Sig.
	Mean ± SD	Mean ± SD			
Age (years)	22.55 ± 1.22	21.82 ± 0.80	2.337	0.024	S
Height (cm)	168.95 ± 8.87	164.59 ± 9	1.621	0.113	NS
Weight (kg)	66.82 ± 7.74	63.95 ± 9.5	1.095	0.280	NS
BMI (kg/m^2^)	23.32 ± 1.38	23.39 ± 1.22	−0.151	0.881	NS

All data presented as Mean ± SD = Standard deviation, t-value = t-statistic, *p*-value = probability, Sig. = Significance, S = Significant, NS = non-significant.

**Table 2:** Effect of time and group × time interaction on dependent variables (repeated measures MANOVA).

Variable	Group × Time Interaction			Effect of Time		
	F-Value	*p*-Value	Partial η^2^	F-Value	*p*-Value	Partial η^2^
VAS	1.42	0.240	0.033	569.34	<0.001 *	0.931
NDI	0.317	0.576	0.007	228.03	<0.001 *	0.844
DNF	11.071	0.002 *	0.209	1284.89	<0.001 *	0.968
CVA	0.258	0.614	0.006	350.00	<0.001 *	0.893

* Significant; VAS: visual analog scale; NDI: neck disability index; DNF: deep neck flexor activation; CVA: craniovertebral angle.

**Table 3:** Mean, within-, and between-group comparisons for all dependent variables in both groups.

Outcome Measure	Group	Pre-Intervention (Mean ± SD)	Post-Intervention (Mean ± SD)	Within-Group MD (CI 95%)	Within-Group *p*-Value	*Sig*.
VAS	Group A	56.227 ± 13.45	14.50 ± 8.71	41.727 (35.75; 47.71)	<0.001 *	S
	Group B	63.84 ± 13.81	17.73 ± 9.34	46.11 (41.33; 50.89)	<0.001 *	S
	Between-Group MD (95% CI)	−7.61 (−15.91; 0.68)	−3.227 (−8.721; 2.267)			
	Between-Group *p*-value	0.071	0.242			
	Sig.	***NS***	***NS***			
NDI	Group A	26.44 ± 7.56	6.77 ± 5.23	19.67 (15.68; 23.65)	<0.001 *	S
	Group B	30.35 ± 10.47	9.16 ± 4.57	21.19 (17.22; 25.16)	<0.001 *	S
	Between-Group MD (95% CI)	−3.91 (−9.47; 1.64)	−2.39 (−5.37; 0.59)			
	Between-Group *p*-value	0.163	0.114			
	Sig.	***NS***	***NS***			
DNF	Group A	23.02 ± 1.52	29.28 ± 0.61	−6.26 (−6.91; −5.61)	<0.001 *	S
	Group B	22.33 ± 1.10	29.87 ± 0.28	−7.54 (−8.01; −7.08)	<0.001 *	S
	Between-Group MD (95% CI)	−0.69 (−0.12; 1.50)	−0.59 (−0.88; −0.31)			
	Between-Group *p*-value	0.093	<0.001 *			
	Sig.	***NS***	S			
CVA	Group A	41.11 ± 4.85	51.19 ± 3.50	−10.08 (−11.47; −8.68)	<0.001 *	S
	Group B	39.93 ± 3.95	49.48 ± 2.59	−9.55 (−11.22; −7.87)	<0.001 *	S
	Between-Group MD (95% CI)	1.17 (−1.51; 3.87)	1.71 (−0.16; 3.58)			
	Between-Group *p*-value	0.382	0.073			
	Sig.	***NS***	***NS***			

SD: Standard deviation; MD: Mean difference; *p*-value: Probability value; S: Significant; NS: Non-significant; * significant; CI: Confidence Interval, VAS: visual analog scale; NDI: neck disability index; DNF: deep neck flexor activation; CVA: craniovertebral angle. Within-group mean differences were calculated as pre-intervention minus post-intervention values. Therefore, negative mean difference values for DNF endurance and CVA indicate improvement following the intervention.

## Data Availability

Material and Data are available upon appropriate request from the corresponding authors.

## Supplementary Materials

The following supporting information can be downloaded at: https://www.mdpi.com/article/10.3390/jcm15124833/s1, Table S1: TREND (Transparent Reporting of Evaluations with Nonrandomized Designs) Checklist for Non-Randomized Intervention Studies.

## Abbreviations

The following abbreviations are used in this manuscript:

NP	Neck pain
NSNP	Nonspecific neck pain
DNF	Deep neck flexor
CVA	Craniovertebral angle
NDI	Neck disability index
NDI-Ar	Neck disability index Arabic version
VAS	Visual analog scale
FHP	Forward head posture
GPR	Global postural re-education
